# A Phosphatase‐Mimetic Nano‐Stabilizer of Mast Cells for Long‐Term Prevention of Allergic Disease

**DOI:** 10.1002/advs.202004115

**Published:** 2021-02-08

**Authors:** Peihua Lin, Mengda Cao, Fan Xia, Hongwei Liao, Heng Sun, Qiyue Wang, Jiyoung Lee, Yan Zhou, Yunan Guan, Cheng Zhang, Zhiqiang Xu, Fangyuan Li, Ji‐Fu Wei, Daishun Ling

**Affiliations:** ^1^ Institute of Pharmaceutics College of Pharmaceutical Sciences Zhejiang University Hangzhou Zhejiang 310058 P. R. China; ^2^ Research Division of Clinical Pharmacology The First Affiliated Hospital Nanjing Medical University Nanjing Jiangsu 210029 P. R. China; ^3^ Hangzhou Institute of Innovative Medicine Zhejiang University Hangzhou Zhejiang 310058 P. R. China; ^4^ Women & Children Central Laboratory The First Affiliated Hospital Nanjing Medical University Nanjing Jiangsu 210036 P. R. China; ^5^ School of Chemistry and Chemical Engineering Frontiers Science Center for Transformative Molecules National Center for Translational Medicine Shanghai Jiao Tong University Shanghai 200240 P. R. China

**Keywords:** allergic disease prevention, ceria nanoparticles, mast cells, phosphatase‐mimetic activity, therapeutic time window

## Abstract

Allergic diseases are pathological immune responses with significant morbidity, which are closely associated with allergic mediators as released by allergen‐stimulated mast cells (MCs). Prophylactic stabilization of MCs is regarded as a practical approach to prevent allergic diseases. However, most of the existing small molecular MC stabilizers exhibit a narrow therapeutic time window, failing to provide long‐term prevention of allergic diseases. Herein, ceria nanoparticle (CeNP‐) based phosphatase‐mimetic nano‐stabilizers (PMNSs) with a long‐term therapeutic time window are developed for allergic disease prevention. By virtue of the regenerable catalytic hotspots of oxygen vacancies on the surface of CeNPs, PMNSs exhibit sustainable phosphatase‐mimetic activity to dephosphorylate phosphoproteins in allergen‐stimulated MCs. Consequently, PMNSs constantly modulate intracellular phospho‐signaling cascades of MCs to inhibit the degranulation of allergic mediators, which prevents the initiation of allergic mediator‐associated pathological responses, eventually providing protection against allergic diseases with a long‐term therapeutic time window.

## Graphical abstract

Ceria nanoparticle (CeNP‐) based phosphatase‐mimetic nano‐stabilizers (PMNSs) of mast cells (MCs) are developed for allergic disease prevention. By virtue of the regenerable catalytic hotspots of oxygen vacancies on the surface of CeNPs, PMNSs can constantly modulate degranulation‐associated phospho‐signaling cascades in allergen‐stimulated MCs via the dephosphorylation of phosphoproteins, enabling the long‐term therapeutic time window for allergic disease prevention.

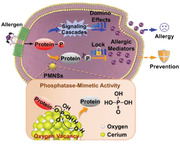

The prevalence of allergic diseases has increased dramatically to epidemic proportions worldwide. More than 25% of the people worldwide suffer from numerous allergic diseases,^[^ ^1^ ^]^ including asthma, rhinitis, atopic dermatitis, food allergies, and even life‐threatening anaphylaxis.^[^ ^2^ ^]^ Unfortunately, 90% of patients with these allergic conditions are insufficiently treated,^[^ ^3^ ^]^ which brings unbearable sufferings to patients and socioeconomic burden. Allergic diseases are pathological immune responses involving an array of immune cells, among which mast cells (MCs) are regarded as principal effector cells.^[^ ^4^ ^]^ Once stimulated by allergens, MCs would activate intracellular phospho‐signaling cascades^[^ ^5^ ^]^ to induce the “domino effects”, including the generation of second messengers such as reactive oxygen species (ROS),^[^ ^6^ ^]^ the degranulation of allergic mediators, and the subsequent pathological responses (e.g., bronchoconstriction, increased vascular permeability, vasodilation, and inflammation), thus leading to the occurrence of allergic diseases.^[^ ^7^ ^]^

Until recently, antihistamines, steroids, and nonsteroidal anti‐inflammatory drugs are the mainstays for combating allergic diseases; however, these drugs can only relieve pathological symptoms rather than prevent the occurrence of allergic diseases.^[^ ^8^ ^]^ Alternatively, MC stabilizers that can inhibit the degranulation of allergic mediators are promising candidates for allergic disease prevention. Nevertheless, the practical usage of most existing small molecular MC stabilizers, including the gold standard disodium cromoglycate (DSCG), is restricted by their narrow therapeutic time window.^[^ ^9^ ^]^ MCs can be stabilized effectively only when these MC stabilizers are administrated immediately before antigenic stimulation. Although nanozymes (nanomaterials with enzyme‐mimetic activities)^[^ ^10^ ^]^ with sustainable enzymatic activities have been demonstrated to alleviate the degranulation of MCs by scavenging ROS,^[^ ^11^ ^]^ none of them thus far were reported to modulate degranulation‐related phospho‐signaling cascades of MCs to directly prevent allergic diseases. Due to the unpredictable nature of allergic diseases, patients with allergic constitution often miss the optimal administration time of DSCG.^[^ ^9a^ ^]^ Therefore, it is crucial to develop efficient MC stabilizers with a long‐term therapeutic time window for the prevention of allergic diseases.

Herein, we report ultrafine ceria nanoparticles (CeNPs) with sufficient surface oxygen vacancies as phosphatase‐mimetic nano‐stabilizers (PMNSs) to efficiently protect against allergic diseases via the stabilization of MCs (**Scheme** **1**). Cerium ion is known to promote the hydrolysis of phosphorylated substrates by cleaving the phosphoester bonds.^[^ ^12^ ^]^ The as‐synthesized CeNPs with abundant surface cerium ions can be readily internalized into MCs, and directly modulate the intracellular phospho‐signaling cascades to stabilize allergen‐stimulated MCs based on their phosphatase‐mimetic activities. The detailed mechanism for the preventive effect of PMNSs on allergic diseases is elucidated, including the critical role of surface oxygen vacancies, the modulating effect on phospho‐signaling cascades, and the stabilizing effect on allergen‐stimulated MCs. In vivo study demonstrates a long‐term therapeutic time window of PMNSs in passive cutaneous anaphylaxis (PCA) mice model, indicating the immense potential of PMNSs to address the unsatisfactory prevention of allergic diseases.

**Scheme 1:**
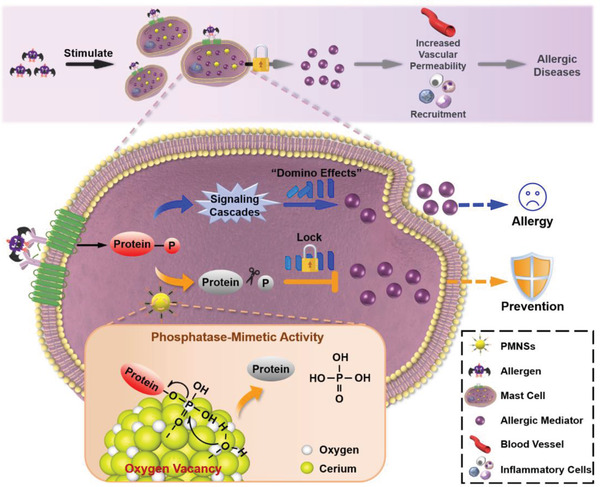
Schematic illustration of PMNSs for allergic disease prevention. By virtue of the regenerable catalytic hotspots of surface oxygen vacancies, PMNSs constantly modulate phospho‐signaling cascades in MCs to lock the “domino effects” of the degranulation of allergic mediators and the initiation of pathological responses via dephosphorylating phosphoproteins, thus enabling the long‐term therapeutic time window for allergic disease prevention.

The ultrafine CeNPs with a diameter of ≈3 nm were fabricated by a modified reverse micelle method,^[^ ^13^ ^]^ as confirmed by the transmission electron microscopy (TEM) image (Figure S1a, Supporting Information) and the X‐ray diffraction (XRD) pattern (Figure S1b, Supporting Information). High‐resolution TEM (HRTEM) image indicates that CeNPs are enriched with surface oxygen vacancies (**Figure** **1**a). To maintain the electrostatic balance around the surface oxygen vacancies of CeNPs, a portion of Ce^4+^ is reduced to Ce^3+^,^[^ ^14^ ^]^ as verified by the X‐ray photoelectron spectroscopy analysis (Figure S1c, Supporting Information). The as‐synthesized CeNPs were further modified with 1,2‐distearoyl‐sn‐glycero‐3‐phosphoethanolamine‐N‐[methoxy(polyethylene glycol)‐2000] (DSPE‐PEG_2000_), acquiring water‐dispersible and stable PMNSs (with a hydrodynamic diameter of ≈9 nm) for further biomedical applications (Figure S2, Supporting Information).^[^ ^15^ ^]^

**Figure 1:**
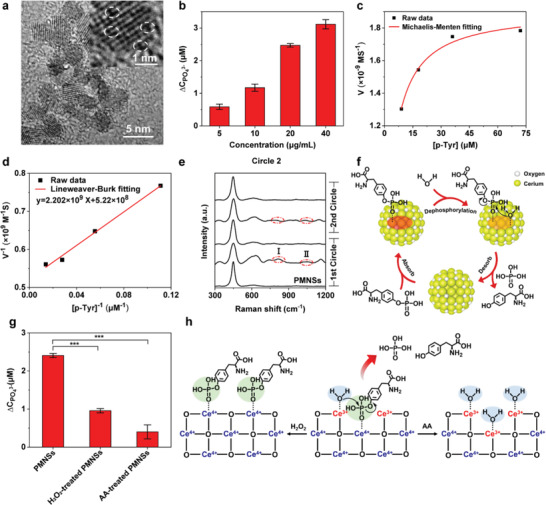
Design and characterization of CeNPs based PMNSs to protect against allergic diseases. a) HRTEM image of CeNPs. Insert, magnified HRTEM image of CeNPs. Dark pits marked by the white dashed circle represent oxygen vacancies. b) The phosphatase‐mimetic activity of PMNSs at different concentrations (*n* = 3). c,d) Michaelis–Menten kinetics (c) and Lineweaver–Burk plotting (d) of PMNSs obtained by adding different concentrations of P‐Tyr. e) Raman spectra of PMNSs undergoing 2 circles of dephosphorylation reaction, indicating the sustainable phosphatase‐mimetic activity of PMNSs. I, the band at ≈830 nm^–1^ is assigned to ring breathing of Tyr and P‐Tyr; II, the band at ≈1070 nm^–1^ is assigned to P‐O stretch vibrations associated with PO_2_^–^ moiety. f) Schematic illustration of the sustainable phosphatase‐mimetic activity of PMNSs. First, P‐Tyr and H_2_O are absorbed on the surface oxygen vacancies of PMNSs for an SN_2_ hydrolysis reaction, and consequently the phosphate monoester bond of P‐Tyr is cleaved. Subsequently, the Tyr and free phosphate are desorbed from the surface of PMNSs, contributing to the regeneration of catalytic hotspots for sustainable phosphatase‐mimetic activity. The red ellipse and orange ellipse represent the forming and cleaving of the weak covalent interaction between cerium ions and the phosphate group of P‐Tyr, respectively. g) The phosphatase‐mimetic activity of PMNSs with different pretreatments (*n* = 3). h) Schematic representation of the oxygen vacancy‐based phosphatase mimetic‐activity of PMNSs. AA, ascorbic acid. Data represent means ± s.e.m. ***p < 0.001.

Since tyrosine phosphorylation is the major event in degranulation‐related phospho‐signaling cascades of allergen‐stimulated MCs,^[^ ^5c,d^ ^]^ we studied the phosphatase‐mimetic activity of PMNSs by using O‐phospho‐L‐tyrosine (P‐Tyr) as the substrate and the produced free phosphate was analyzed by using a malachite green assay. The results show that PMNSs can hydrolyze the phosphate monoester bond of P‐Tyr in a concentration‐dependent manner, demonstrating the phosphatase‐mimetic activity of PMNSs (Figure 1b and Figure S3: Supporting Information). According to the Michaelis–Menten plot and Lineweaver–Burk plot (Figure 1c,d), the Michaelis–Menten constant (*K*_M_) as well as the maximum velocity (*V*_max_) of PMNSs are 4.23 × 10^−6^ m and 1.92 × 10^–9^ m s^–1^, respectively. PMNSs retain the cubic fluorite structure during the hydrolytic process as indicated by XRD patterns (Figure S4, Supporting Information), confirming the excellent stability of PMNSs. Moreover, the dephosphorylation reaction occurred on the surface of PMNSs as indicated by Raman spectra analysis (Figure 1e).^[^ ^16^ ^]^ It is noteworthy that the P‐O stretch vibration associated with phosphodioxy (PO_2_^–^) moiety disappears on the surface of PMNSs after the dephosphorylation reaction, indicating Tyr and free phosphate are desorbed from the surface of PMNSs, which contributes to the regeneration of catalytic hotspots for the next circle of dephosphorylation reaction (Figure 1f). All these results demonstrate the sustainable and excellent phosphatase‐mimetic activity of PMNSs. Moreover, with the reversible switch between Ce^3+^ (reduction) and Ce^4+^ (oxidation),^[^ ^17^ ^]^ PMNSs also exhibit prominent superoxide dismutase‐mimetic, catalase‐mimetic activities and the hydroxyl radical antioxidant capacity for ROS scavenging (Figure S5, Supporting Information). Increasing evidences show that surface oxygen vacancies are tightly associated with the enzyme‐mimetic activities of CeNPs.^[^ ^14^, ^18^ ^]^ To elucidate the catalytic mechanism of phosphatase‐mimetic activity, we treated PMNSs with H_2_O_2_ to oxidize Ce^3+^ to Ce^4+^ or with ascorbic acid (AA) to reduce Ce^4+^ to Ce^3+^,^[^ ^19^ ^]^ which subsequently disturbed the electrostatic balance and decreased the number of surface oxygen vacancies. As a result, the phosphatase‐mimetic activity of PMNSs is inhibited under both treatments (Figure 1g), indicating the Ce^3+^ and Ce^4+^ around the surface oxygen vacancies of PMNSs can bind with H_2_O and P‐Tyr respectively for an SN_2_ hydrolysis reaction (Figure 1h).^[^ ^12a,b^ ^]^

PMNSs with phosphatase‐mimetic activity are expected to stabilize MCs by modulating intracellular phospho‐signaling cascades (**Figure** **2**a). Firstly, PMNSs can be readily internalized into mouse bone marrow‐derived MCs (BMMCs) (Figure 2b and Figure S6: Supporting Information), and retain in BMMCs (Figures S7 and S8, Supporting Information) without inducing any evident cytotoxicity or pseudoallergic reaction (Figures S9 and S10, Supporting Information), ensuring the long‐term prevention of allergic diseases. Moreover, the exocytosis of PMNSs through lysosome secretion indicates the excellent biosafety of PMNSs (Figure S7, Supporting Information).^[^ ^20^ ^]^ We further treated BMMCs with PMNSs or DSCG at indicated time points prior to antigenic stimulation, followed by measuring the release level of *β*‐hexosaminidase (*β*‐HEX) to assess the MC stabilizing effect.^[^ ^7a^ ^]^ Consistent with the previous report,^[^ ^9a,b^ ^]^ DSCG exhibits MC stabilizing effect only when administrated immediately prior to antigenic stimulation, while PMNSs demonstrate a long‐term therapeutic time window for stabilizing BMMCs (Figure 2c and Figure S11: Supporting Information).

**Figure 2:**
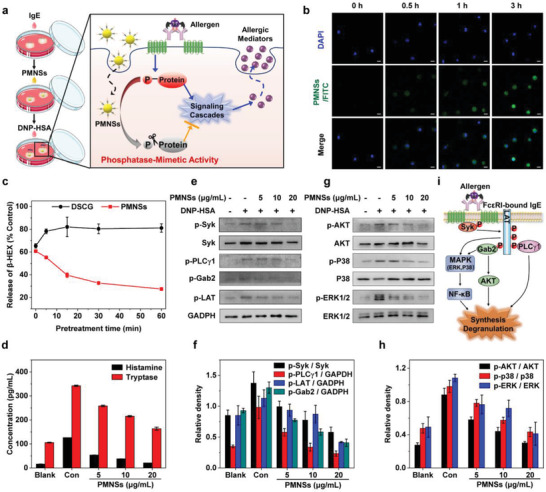
Mast cell stabilizing effect of PMNSs in vitro. a) Schematic illustration of the experimental setup to evaluate the MC stabilizing effect of PMNSs and the mechanism of PMNSs in stabilizing allergen‐stimulated BMMCs. PMNSs can dephosphorylate phosphoproteins, and thus inhibit the intracellular phospho‐signaling cascades to stabilize allergen‐stimulated BMMCs. b) Representative confocal laser scanning microscopic images of the BMMCs incubated with PMNSs/FITC for various time points. Scale bar: 10 µm. c) The release level of *β*‐HEX of BMMCs pretreated with PMNSs or DSCG for different time. Control group was stimulated by DNP‐HSA without the treatment of DSCG or PMNSs. The *β*‐HEX release of the control group is 26.6 ± 1.1% of the total contents (*n* = 3). d) The release levels of histamine and tryptase of BMMCs pretreated with different concentrations of PMNSs (*n* = 3). e,f) Western blot analysis (e) and the relative phosphorylation levels (f) of Syk, PLC*γ*1, Gab2, and LAT in Fc*ε*RI‐mediated signaling cascades of BMMCs with different treatments (*n* = 3). g,h) Western blot analysis (g) and the relative phosphorylation levels (h) of AKT, p38, and ERK1/2 in downstream pathways of Fc*ε*RI‐mediated signaling cascades of BMMCs with different treatments (*n* = 3). The intensity of the bands was determined using densitometric analysis. i) Schematic illustration of intracellular degranulation‐related phospho‐signaling cascades of MCs. The crosslinking between two Fc*ε*RI‐bound IgEs induced by allergen stimulation activates MCs to initiate intracellular phospho‐signaling cascades, leading to the synthesis and degranulation of allergic mediators. Data represent means ± s.e.m.

The release of histamine and tryptases that are closely associated with allergic reactions is remarkably inhibited in PMNSs pretreated BMMCs (Figure 2d). As the crosslinking between allergens and Fc epsilon receptor I (Fc*ε*RI)‐bound immunoglobulin E (IgE) could activate MCs to initiate intracellular degranulation‐related phospho‐signaling cascades,^[^ ^21^ ^]^ we further evaluated the phosphorylation levels of key protein kinases in Fc*ε*RI‐mediated signaling cascades of MCs, including spleen tyrosine kinase (Syk), phospholipase C*γ*1 (PLC*γ*1), GRB2‐associated binding protein 2 (Gab2), and linker for activation of T cells (LAT). Notably, the phosphorylation levels of the aforementioned proteins in PMNSs pretreated BMMCs decrease as compared with the control group, indicating the modulating effect of PMNSs on phospho‐signaling cascades of MCs (Figure 2e,f). Furthermore, PMNSs reduce the phosphorylation levels of the allergic mediator synthesis‐related downstream signaling of Fc*ε*RI‐mediated signaling cascades in MCs, including protein kinase B (AKT), extracellular signal‐regulated kinase 1/2 (ERK1/2), p38 mitogen‐activated protein kinase (p38 MAPK, p38), I*κ*B kinase *α*/*β* (IKK*α*/*β*) and nuclear factor‐*κ*B (NF‐*κ*B) (Figure 2g,h and Figure S12: Supporting Information).^[^ ^5^, ^22^ ^]^ Moreover, since the production of ROS is activated by the initiation of degranulation‐related phospho‐signaling cascades,^[^ ^6^ ^]^ PMNSs with phosphatase‐mimetic activity and antioxidant activity can significantly reduce the ROS level of allergen‐stimulated BMMCs (Figure S13, Supporting Information). These collective results demonstrate that PMNSs can efficiently modulate phospho‐signaling cascades of MCs to prevent allergic diseases.

To evaluate the long‐term MC stabilizing effect of PMNSs in vivo, the IgE‐sensitized mice were treated with PMNSs or DSCG 6 h before the PCA reaction induced by antigenic stimulation (**Figure** **3**a).^[^ ^23^ ^]^ The allergic mediators released by allergen‐stimulated MCs increase the vascular permeability in allergic tissues, leading to the extravasation of Evans blue dye (Figure 3b).^[^ ^24^ ^]^ As demonstrated by the leakage of Evans blue dye in allergen‐stimulated mice, DSCG fails to inhibit the PCA reaction, while PMNSs substantially protect against PCA reaction (Figure 3c,d), indicating the long‐term therapeutic time window of PMNSs for allergic diseases. Toluidine blue staining of skin sections shows that MCs of PMNSs pretreated mice are full of secretory granules, while MCs of DSCG pretreated mice are significantly degranulated (Figure 3e).^[^ ^25^ ^]^ Moreover, the allergic mediators released by MCs recruit and activate inflammatory cells into the allergic tissues to aggravate allergic diseases.^[^ ^7^, ^26^ ^]^ Compared with DSCG pretreated mice, the recruitment of inflammatory cells is markedly inhibited in PMNSs pretreated mice (Figure 3e), indicating the suppressed allergic reaction. Furthermore, the histopathological examinations show no pathological change in heart, liver, spleen, lungs, kidneys, and skins (Figures S14 and 15, Supporting Information). All these in vivo results indicate that the highly biocompatible PMNSs can serve as potential MC stabilizers with a long‐term therapeutic time window.

**Figure 3:**
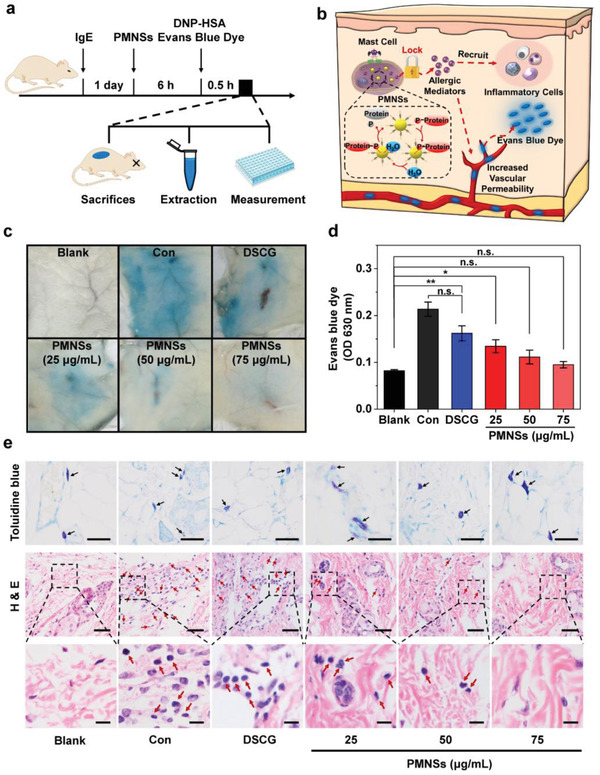
Preventive effect of PMNSs on allergic diseases in vivo. a) Experimental scheme of PCA mice model establishment, pretreatment schedule, and preventive effect assessments. b) Schematic illustration of PMNSs that protect against allergic diseases with a long‐term therapeutic time window. Benefitting from the sustainable phosphatase‐mimetic activity, PMNSs can constantly stabilize allergen‐stimulated MCs to prevent the increasement of vascular permeability and recruitment of inflammatory cells, thus protecting against allergic diseases with a long‐term therapeutic time window. c,d) Representative photographs (c) and quantitative analysis (d) of Evans blue dye leakage caused by the PCA reaction on the dorsal skin (*n* = 3). e) Representative toluidine blue and hematoxylin and eosin (H&E) staining images of dorsal skin sections obtained from BALB/c mice after various treatments. MC granules stained with toluidine blue were indicated by black arrows; inflammatory cells stained with H&E were indicated by red arrows. Scale bar: 10 µm, top; 10 µm, meddle; 2.5 µm, bottom. Data represent means ± s.e.m. *p < 0.05, **p < 0.01, n.s. no significance.

In summary, CeNPs based PMNSs with a long‐term therapeutic time window are developed for highly efficient allergic disease prevention. The regenerable catalytic hotspots of surface oxygen vacancies endow PMNSs with sustainable and excellent phosphatase‐mimetic activity. Therefore, PMNSs can inhibit the degranulation‐related phospho‐signaling cascades in allergen‐stimulated MCs, and thus inhibit the degranulation of allergic mediators that could induce numerous pathological responses. Subsequently, PMNSs successfully stabilize MCs in vitro and in vivo in a long‐term manner, demonstrating that PMNSs can be utilized as promising MC stabilizers to address the unsatisfied allergic disease prevention.

## Conflict of Interest

The authors declare no conflict of interest.

## Supporting information

Supporting Information

## Data Availability

The data that support the findings of this study are available from the corresponding author upon reasonable request.
