# Supplementary material for: A Phosphatase‐Mimetic Nano‐Stabilizer of Mast Cells for Long‐Term Prevention of Allergic Disease
Source: Adv Sci (Weinh). 2021 Feb 8;8(8):2004115. doi: 10.1002/advs.202004115 (PMC8061383; doi:10.1002/advs.202004115)
Supplement: Supplementary file 1 — Supporting Information [file ADVS-8-2004115-s001.pdf]

WILEY-VCH

© 2021 Wiley-VCH GmbH

ADVANCED SCIENCE
Open Access

Supporting Information

for *Adv. Sci.*, DOI: 10.1002/advs.202004115

A Phosphatase-Mimetic Nano-Stabilizer of Mast Cells for Long-Term Prevention of Allergic Disease

Peihua Lin, Mengda Cao, Fan Xia, Hongwei Liao, Heng Sun, Qiyue Wang, Jiyoung Lee, Yan Zhou, Yunan Guan, Cheng Zhang, Zhiqiang Xu, Fangyuan Li, Ji-Fu Wei*, Daishun Ling**

Supporting Information

A Phosphatase-Mimetic Nano-Stabilizer of Mast Cells for Long-Term Prevention of Allergic Disease

Peihua Lin, Mengda Cao, Fan Xia, Hongwei Liao, Heng Sun, Qiyue Wang, Jiyoung Lee, Yan Zhou, Yunan Guan, Cheng Zhang, Zhiqiang Xu, Fangyuan Li, Ji-Fu Wei*, Daishun Ling**

Prof. D. Ling, Prof. F. Li, P. Lin,[+] F. Xia, H. Liao, H. Sun, Q. Wang, J. Lee, Y. Zhou, Y. Guan
Institute of Pharmaceutics, College of Pharmaceutical Sciences, Zhejiang University, Hangzhou, Zhejiang 310058, P. R. China
E-mail: lingds@zju.edu.cn (Prof. D. Ling), lfy@zju.edu.cn (Prof. F. Li)

Prof. J.F. Wei, M. Cao,[+] Z. Xu
Research Division of Clinical Pharmacology, The First Affiliated Hospital, Nanjing Medical University, Nanjing, Jiangsu 210029, P. R. China
E-mail: weijifu@hotmail.com (Prof. J.F. Wei)

Prof. D. Ling, Prof. F. Li, F. Xia
Hangzhou Institute of Innovative Medicine, Zhejiang University, Hangzhou, Zhejiang 310058, P. R. China

C. Zhang
Women & Children Central Laboratory, The First Affiliated Hospital, Nanjing Medical University, Nanjing, Jiangsu 210036, P. R. China

Prof. D. Ling, Prof. F. Li
Key Laboratory of Biomedical Engineering of the Ministry of Education, College of Biomedical Engineering & Instrument Science, Zhejiang University, Hangzhou, Zhejiang 310058, P. R. China

[+] These authors contributed equally to this work.

Keywords: ceria nanoparticles; phosphatase-mimetic activity; mast cells; therapeutic time window; allergic disease prevention

Supporting methods

Materials. All reagents and solvents were obtained commercially and used without further purification. Oleylamine, xylene, and dinitrophenyl-human serum albumin (DNP-HSA) were purchased from Sigma-Aldrich Co. (St. Louis, MO, USA). Cerium acetate hydrate, *O*-phospho-L-tyrosine (P-Tyr), ascorbic acid, and potassium phosphate monobasic (KH_2PO_4) were purchased from Aladdin Industrial Inc. (Shanghai, China). Hydrogen peroxide (H_2O_2), hexane, and chloroform were purchased from Sinopharm Co. (China). DSPE-mPEG$_{2000}$ was purchased from Shanghai Advanced Vehicle Technology Pharmaceutical Co., Ltd. (Shanghai, China). RPMI 1640 medium, fetal bovine serum, and penicillin-streptomycin mixture were purchased from Gibco (USA). IL-3 and stem cell factor were purchased from Peprotech. Antibodies used in western blot were all purchased from Cell Signaling Technology.

Instruments. Transmission electron microscopy (TEM) was taken to observe the morphology of CeNPs and PMNSs (Hitachi HT7700, Tokyo, Japan) at a voltage of 100 kV. High-resolution TEM was taken with a FEI Tecnai F20 (FEI, USA) at a voltage of 200 kV. The concentration of Ce was quantified by using ICP-MS (PerkinElmer NexION 300X). The hydrodynamic size of PMNSs was detected by using Zetasizer Nano ZS90 (Malvern Instruments, Worcestershire, UK). The X-ray powder diffraction (XRD) pattern was obtained via a Rigaku D/Max-2550 PC instrument (Rigaku, Japan). X-ray photoelectron spectroscopy (XPS) spectrum was obtained via a Thermo Scientific ESCALAB 250 Xi XPS system. Raman spectra were obtained via LabRAM HR evolution (HORIBA, France).

Synthesis of ultrafine ceria nanoparticles (CeNPs). CeNPs were fabricated via a

modified reverse micelle method.[1] Briefly, 0.43 g of cerium acetate hydrate and 3.25 g of oleylamine were dissolved in 15 mL of xylene in a three-neck reaction flask, followed by vigorously stirring at room temperature for 12 h. Subsequently, the mixture was heated to 90 ℃ with a heating rate of 2 ℃/min under the argon atmosphere. Then, 1 mL of deionized water was rapidly injected into the heated mixture. After aging at 90 ℃ for 3 h, the mixed solution was cooled to room temperature and 100 mL of ethanol was added to precipitate CeNPs. CeNPs were collected via centrifuge and dispersed in chloroform for further use.

Synthesis of DSPE-mPEG$_{2000}$ modified phosphatase-mimetic nano-stabilizers (PMNSs). Firstly, 1 mL of CeNPs was added into 5 mL of $CHCl_3$ containing 20 mg of DSPE-mPEG$_{2000}$. Then, the mixed solution was evaporated by the rotary evaporator and incubated at 60 ℃ in vacuum for 1 h to remove the chloroform. Then, 2 mL of deionized water was added to obtain DSPE-mPEG$_{2000}$ modified CeNPs that were used as PMNSs for further biological application.

Synthesis of DSPE-mPEG$_{2000}$-FITC modified PMNSs (PMNSs/FITC). Firstly, 1 mL of CeNPs was added into 5 mL of $CHCl_3$ containing 16 mg of DSPE-mPEG$_{2000}$ and 4 mg of DSPE-mPEG$_{2000}$-FITC. Then, the mixed solution was evaporated by the rotary evaporator and incubated at 60 ℃ in vacuum for 1 h to remove the chloroform. Next, 2 mL of deionized water was added to obtain PMNSs/FITC that were used to study the uptake ability of bone marrow-derived mast cells (BMMCs).

Catalase (CAT)-mimetic activity assay. In a typical test, 64 μL of PMNSs (0.625, 1.25, 2.5, 5 mg/mL) and 800 μL of 30% H_2O_2 solution were added into deionized water with a total

volume of 8 mL. The generated O_2 (unit: mg/L) at different reaction times were measured via an oxygen electrode on Dissolved Oxygen Meter JPB-607 (Shanghai INESA Scientific Instrument, China).

Superoxide dismutase (SOD)-mimetic activity assay. The SOD-mimetic activity of PMNSs at various concentrations (5, 10, 20, and 40 μg/mL) was conducted by a Total Superoxide Dismutase Assay Kit with WST-8 (Beyotime, China).

Hydroxyl radical antioxidant capacity (HORAC) assay. The HORAC of PMNSs at different concentrations (5, 10, 20, and 40 μg/mL) was assessed by a hydroxyl free radical assay kit (Nanjing Jiancheng Bioengineering Institute, China).

Phosphatase-mimetic activity assay. The phosphatase-mimetic activity of PMNSs at different Ce concentrations (5, 10, 20, and 40 μg/mL) or different P-Tyr concentrations (9, 18, 36, and 72 μM) was detected by the malachite green assay (Sangon Biotech Co., Ltd., China). The absorbance of the malachite green solution containing P-Tyr was used as blank. A phosphate standard curve was obtained by using KH_2PO_4 as the substrate.

Isolation and culture of bone marrow-derived mast cells. BMMCs were isolated via a previously reported procedure.[2] Briefly, the bone marrow cells from femora and tibiae of 6 to 8 weeks-old female BALB/c mice were cultured in complete RPMI 1640 medium supplemented with IL-3 (10 ng/ml), stem cell factor (10 ng/ml) (Peprotech), Penicillin (100 U/mL), Streptomycin (100 U/mL), non-essential amino acids (0.1 mM) and L-glutamine (2 mM). After at least 4 weeks of culture, the phenotype of BMMCs was assessed by the toluidine blue method.

***In vitro* cell uptake assay.** BMMCs (10^6 cells/mL) were seeded on slides for confocal laser scanning microscopy (CLSM) imaging. BMMCs were incubated with PMNSs/FITC (20 μg/mL) at 37 °C for 0, 0.5, 1, 3, 20 h, respectively. Then, the cells were washed with PBS for three times and fixed in 4% paraformaldehyde for 20 min. The nuclei were stained with DAPI. BMMCs were deposited on slides using Fluorescence Mounting Medium (DAKO). Subsequently, BMMCs were observed by a Leica confocal microscope (ZEISS, LSM 880, Germany) to confirm the cellular uptake. Furthermore, flow cytometry was used to quantitatively evaluate the uptake efficiency of PMNSs.

Retention and exocytosis of PMNSs. BMMCs (10^6 cells/mL) were deposited on the glass bottom culture plates (NEST Biotechnology, China) for CLSM imaging. BMMCs were incubated with PMNSs/FITC (20 μg/mL) at 37 °C for 3 h, and then transferred to fresh medium after being washed and incubated for 0, 24, 48 h, respectively. Lysotracker-red was used to stain the lysosomes for another 20 min. Subsequently, the cells were washed and photographed under a Leica confocal microscope (ZEISS, LSM 880, Germany).

Characterization of the intracellular PMNSs. BMMCs were incubated with PMNSs (20 μg/mL) at 37 °C for 3 h, and then transferred to fresh medium after being washed and incubated for 48 h. Then, the cells were collected via centrifuge (500 x g). The collected BMMCs were washed and treated with Triton X-100 that can dissolve the membrane of BMMCs to release the intracellular PMNSs. The morphology of PMNSs was characterized by using transmission electron microscopy.

Cytotoxicity assay. PMNSs were dissolved and diluted in the culture medium. BMMCs suspension (10^6 cells/mL) was cultured in 96-well plates (100 μL/well) and treated with

sequential concentrations of PMNSs (0, 5, 10, 15, 20, 30 μg/mL) for 24 h or treated with PMNSs (20 μg/mL) for different time (0, 6, 12, 24, 36, 48 h) at 37 ℃ and 5% CO_2 atmosphere to investigate the biocompatibility. The cell viability of BMMCs was measured by adding cell counting kit-8 to each well for 2 h before the absorbance measurement at 450 nm on a microplate reader (Bio Tech, USA). The absorbance of PMNSs was measured in cell-free wells as control.

Degranulation experiment. BMMCs were sensitized with anti-DNP IgE (500 ng/mL) in RPMI 1640 medium overnight at 37 °C. Then, BMMCs were seeded in a 96-well plate (10^6 cells/mL) and treated with PMNSs (20 μg/mL) or disodium cromoglycate (DSCG) (400 μg/mL) for indicated time points (0, 5, 10, 15 min), followed by challenged with DNP-HSA (100 ng/mL) for 1 h. The supernatant of each well was then collected (4 ℃, 12000 r/min, 10 min) and transferred into new wells (50 μL/well).

Degranulation was determined by measuring the release level of β-hexosaminidase (β-HEX). The supernatant was incubated with an equal volume of substrate solution (1.29 mM p-nitrophenyl-N-acetyl-β-D-glucosaminide in 0.1M citrate buffer (pH=4.5)) for 1.5 h at 37 °C. The enzyme reaction was stopped by the addition of 75 μL of glycine buffer (0.2 M, pH 10.7) and the reaction product was measured at 405 nm on a microplate reader (Bio Tech, USA). Untreated cells were used as negative control. Moreover, the concentration of histamine (ab213975, Abcam) and tryptases (CSB-E14326m, Cusabio) was measured with the commercially available enzyme-linked immunosorbent assay kits.

Cellular ROS-scavenging capability of PMNSs. BMMCs (10^6 cells/well) were

sensitized with anti-DNP IgE (500 ng/mL) in culture medium overnight at 37 °C. Then, BMMCs were treated with PMNSs (20 μg/mL) for 3 h and DCFH-DA for 20 min. Subsequently, BMMCs were challenged with DNP-HSA (100 ng/mL) for 1 h in the cell culture incubator before being fixed in 4% paraformaldehyde for 20 min. The nuclei were stained with DAPI. BMMCs were deposited on slides using Fluorescence Mounting Medium (DAKO). The ROS-scavenging capability of PMNSs was monitored by confocal microscopy (ZEISS, LSM 880, Germany). Furthermore, flow cytometry was used to quantitatively evaluate the ROS-scavenging capability of PMNSs.

Western blot analysis. BMMCs were sensitized with anti-DNP IgE (500 ng/mL) in RPMI 1640 medium and cultured at 37 °C overnight. Then, BMMCs were cultured with PMNSs at different concentrations (0, 5, 10, 20 μg/mL) for 1 h and then challenged with DNP-HSA (100 ng/mL) for 1 h. The cell lysates were collected and analyzed by electrophoresis. The proteins were quantified by using the BCA™ Protein Assay Kit (Sangon Biotech, Shanghai, China) and isolated by 12% sodium dodecyl sulfate-polyacrylamide gel electrophoresis. After being transferred to polyvinylidene fluoride membranes (Millipore, USA) and blocked by 5% nonfat milk, the membranes were incubated at 4 °C overnight with antibodies against p-Syk, Syk, p-PLCγ1, PLCγ1, p-LAT, GAPDH, p-AKT, AKT, p-p38, p38, p-ERK1/2, ERK1/2, p-IKKα/β, IKKα/β, p-NF-κB, and NF-κB, respectively. Then the membranes were washed for 3 times in TBST (Tris-HCl+Tween) and incubated with secondary antibodies for 1 h at room temperature. After being rinsed for 3 times in TBST again, the membranes were probed using immobilon Western chemiluminescent horseradish peroxidase substrate (Millipore, USA) and autoradiographed. The intensity of the bands was determined by densitometric analysis.

Passive cutaneous anaphylaxis (PCA) mice model and *in vivo* prevention. 7-8 week-old female BALB/c mice obtained from Shanghai SLAC Laboratory Animal Co. Ltd. (Shanghai, China) were passively sensitized by intradermal injection of anti-DNP IgE (120 ng/site) in dorsal skin. 24 h later, mice with IgE-sensitized dorsal skin were administrated with PMNSs (25, 50, 75 μg/mL, 20 μL/site) or DSCG (100 μg/mL, 20 μL/site) by intradermal injection 6 h before the intravenous injection of DNP-HSA (1 mg/mL in saline containing 0.5% Evans blue, 200 μL). 30 min later, mice were sacrificed for analysis. Evans blue dye in the IgE-sensitized sites was extracted by formamide (overnight, 65 ℃), and quantified with a microplate reader. Animal experiments were performed according to institutional guidelines and were approved by the Institutional Animal Care and Use Committee of Zhejiang University School of Medicine.

Histological analysis. Dorsal skin tissues from the PCA mice were fixed in 10% formalin for 24 h followed by processing and paraffin embedding. Sections (4 μm) of paraffin-embedded tissues were stained with toluidine blue and hematoxylin and eosin (H&E).

Statistical analysis. Data analysis was performed by using the Origin 2018 (OriginLab Corporation, Northampton, Massachusetts, USA). Statistical significance was determined by Student’s t-test.

References

[1] C. K. Kim, T. Kim, I.-Y. Choi, M. Soh, D. Kim, Y.-J. Kim, H. Jang, H.-S. Yang, J. Y.

Kim, H.-K. Park, S. P. Park, S. Park, T. Yu, B.-W. Yoon, S.-H. Lee, T. Hyeon, *Angew. Chem. Int. Ed.* **2012**, *51*, 11039.

[2] P. Volná, P. Lebduska, L. Dráberová, S. Símová, P. Heneberg, M. Boubelík, V. Bugajev, B. Malissen, B. S. Wilson, V. Horejsí, M. Malissen, P. Dráber, *J. Exp. Med.* **2004**, *200*, 1001.

Supporting figures

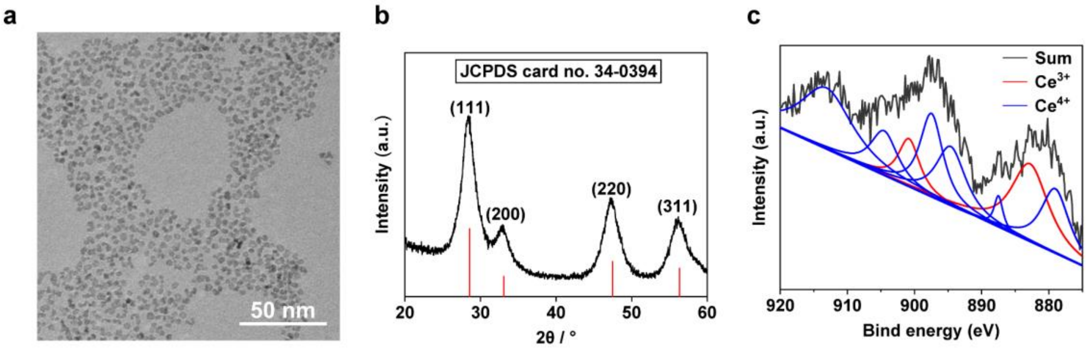

Figure S1. Characterization of CeNPs. a) TEM image of CeNPs. b) XRD pattern of CeNPs, indicating the cubic fluorite structure of CeNPs (JCPDS card no. 34-0394). c) XPS spectrum of CeNPs, confirming the coexistence of Ce^{3+} (BE at 882.8 and 900.8 eV) and Ce^{4+} (BE at 879.0, 887.5, 894.6, 897.5, 904.5, and 913.1 eV) on the surface of CeNPs.

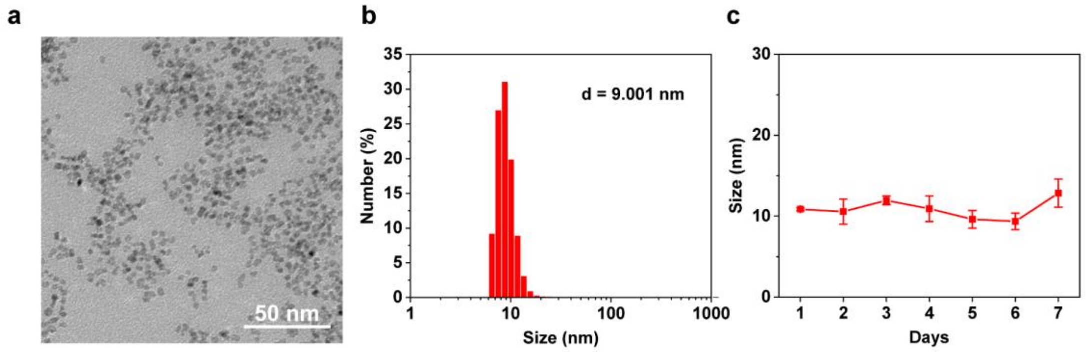

Figure S2. Characterization of PMNSs. a) TEM image of PMNSs in water. b) Hydrodynamic diameter distribution of PMNSs in water. c) The colloidal stability of PMNSs in water for 7 days (n = 3). Data represent means ± s.e.m.

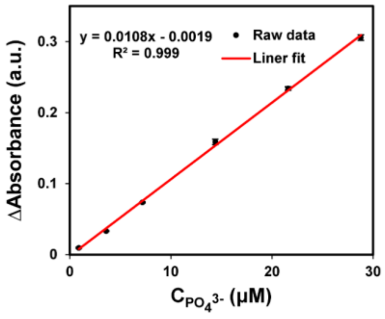

Figure S3. Standard curve of phosphate (n = 5). Data represent means ± s.e.m.

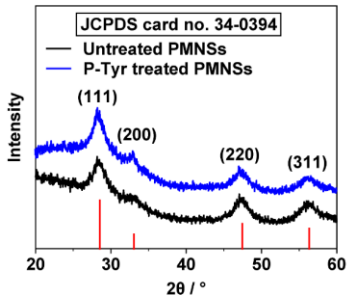

Figure S4. XRD patterns of untreated PMNSs and P-Tyr treated PMNSs.

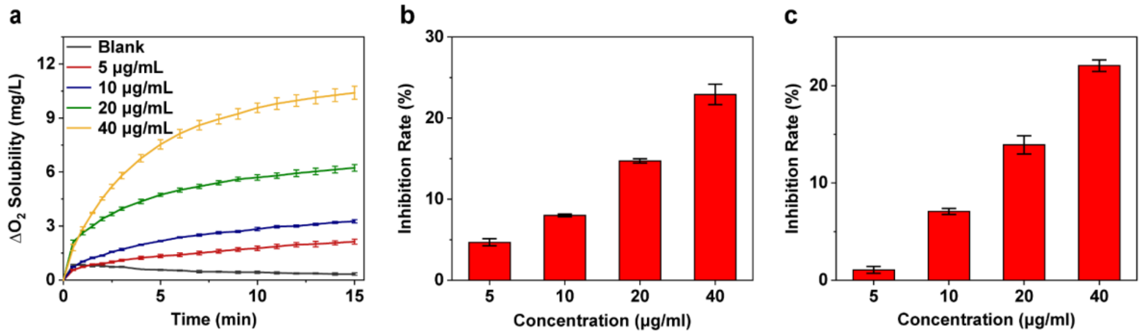

Figure S5. a-c) The antioxidant activities of PMNSs. The CAT-mimetic activity (a), SOD-mimetic activity (b) and HORAC (c) of PMNSs, indicating the excellent ROS scavenging capability of PMNSs (n = 3). Data represent means ± s.e.m.

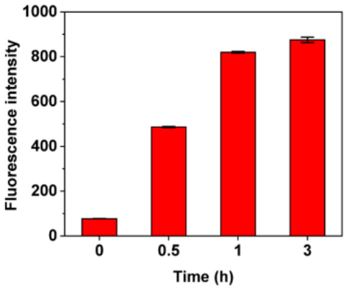

Figure S6. Quantitative measurement of FITC signals in BMMCs to evaluate the uptake efficiency of PMNSs/FITC via flow cytometry (n = 3). Data represent means ± s.e.m.

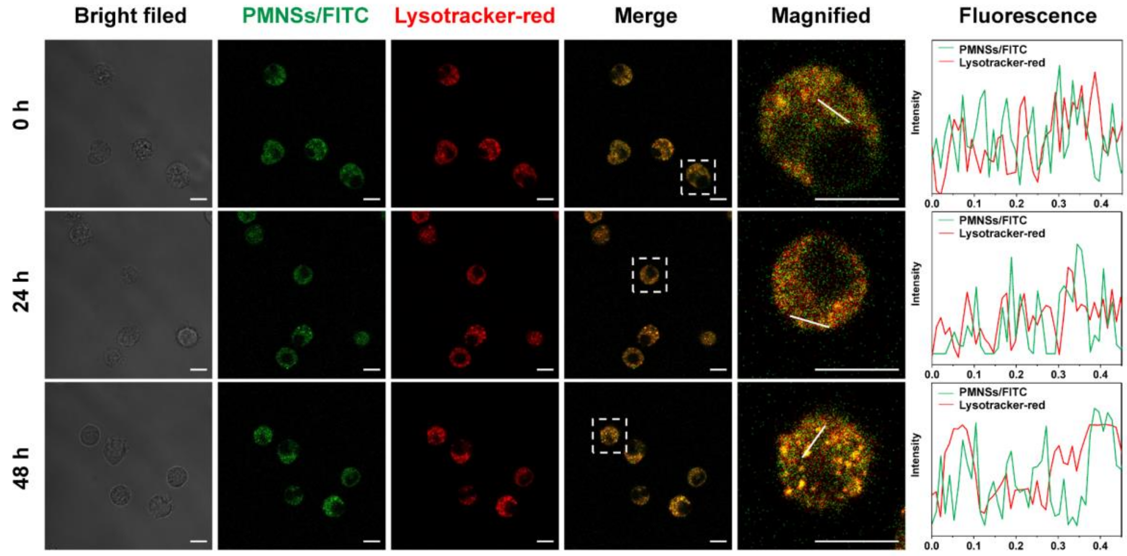

Figure S7. Representative CLSM images of the PMNSs/FITC incubated BMMCs and co-localization analysis of PMNS/FITC (green) and lysosome (red). To track the location of PMNSs within BMMCs, PMNSs were labeled with FITC. BMMCs were incubated with PMNSs/FITC for 3 h, and then incubated in fresh medium for different times. The overlapping fluorescent signals of green fluorescence from PMNSs/FITC and red fluorescence from lysosome increase in a time dependent manner, indicating PMNSs can be excluded from BMMCs via lysosome secretion.

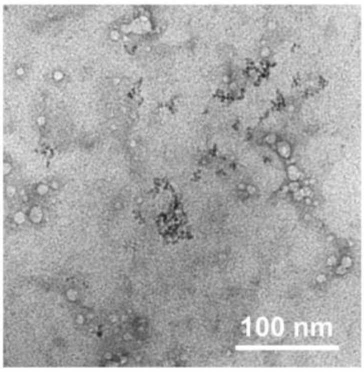

Figure S8. TEM image of intracellular PMNSs. PMNSs incubated BMMCs were treated with Triton X-100 that can dissolve the membrane of BMMCs to release the intracellular PMNSs.

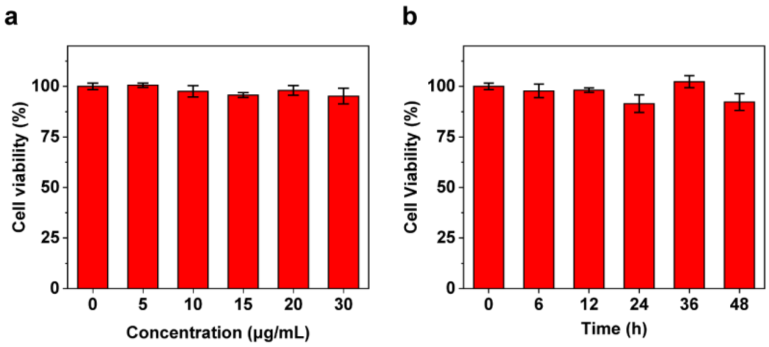

Figure S9. a,b) Cell viability of BMMCs after treated with different PMNSs concentrations for 24 h (a) or treated with PMNSs (20 μg/mL) for different incubation time (b) (n = 3). Data represent means ± s.e.m.

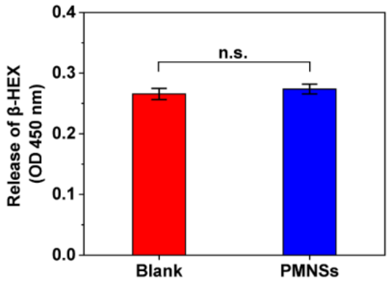

Figure S10. The release level of β-HEX of BMMCs treated with or without CeNPs (n = 3).

Data represent means ± s.e.m.; n.s. no significance.

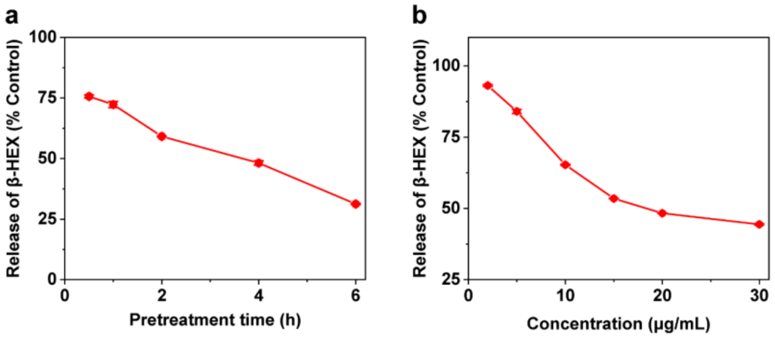

Figure S11. a) The release level of β-HEX of BMMCs pretreated with PMNSs for different time points. The β-HEX release of the control group is 38.1 ± 2.3% of the total contents (n = 3). b) The release level of β-HEX of BMMCs pretreated with varying concentrations of PMNSs for 1h. The β-HEX release of the control group is 76.2 ± 0.5% of the total contents (n = 4). Control group was stimulated by DNP-HSA without the treatment of DSCG or PMNSs. Data represent means ± s.e.m.

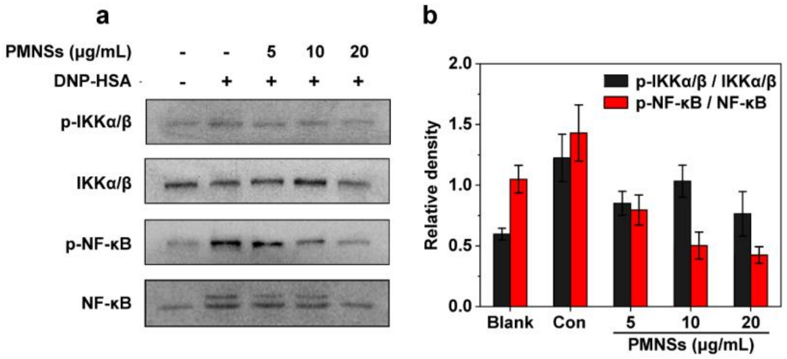

Figure S12. a,b) Western blot analysis (a) and the relative phosphorylation levels (b) of NF-κB and IKKα/β in downstream pathways of FcεRI-mediated signaling cascades of BMMCs with different treatments (n = 3). Data represent means ± s.e.m.

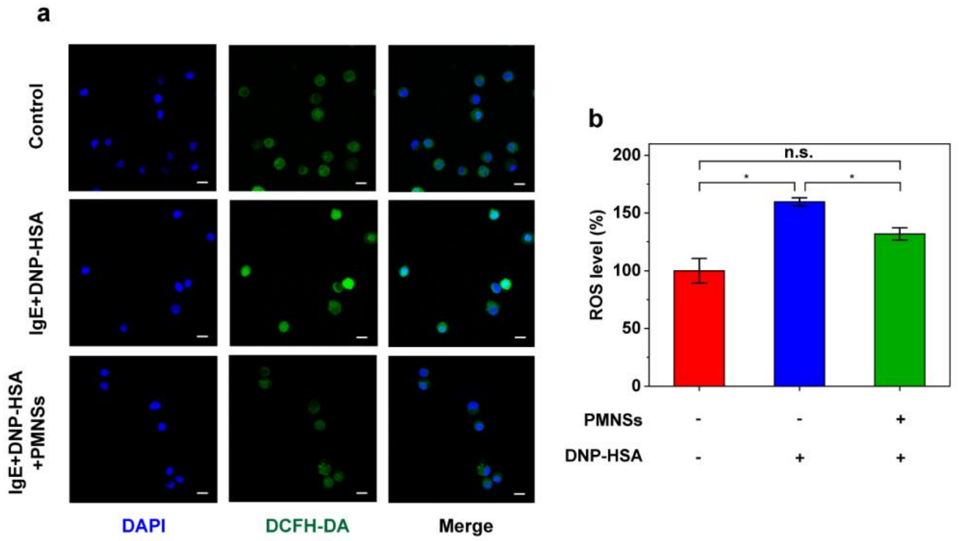

Figure S13. a) Representative CLSM images of ROS formation of BMMCs with different treatments. b) Quantitative measurement of DCFH-DA signals in BMMCs with different treatments by using flow cytometry (n = 3). Data represent means ± s.e.m.; *P < 0.05; n.s. no significance.

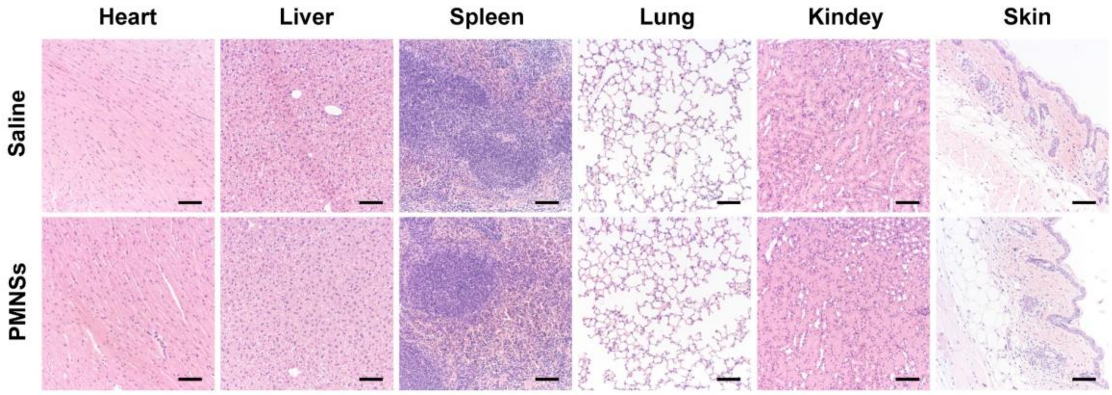

Figure S14. Representative H&E staining images of tissues obtained from different organs at 15 days post-injection of PMNSs. Scale bar: 400 μm.

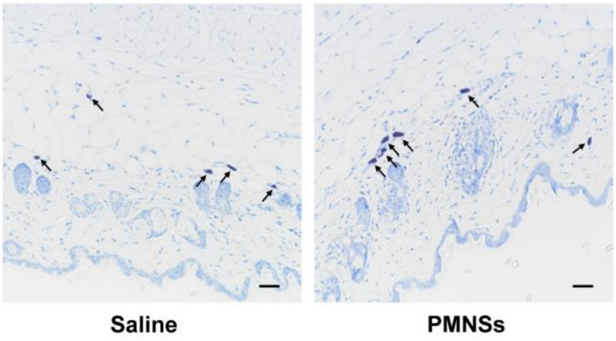

Figure S15. Representative toluidine blue staining images of dorsal skin sections obtained at 15 days post-injection of PMNSs. Scale bar: 200 μm.

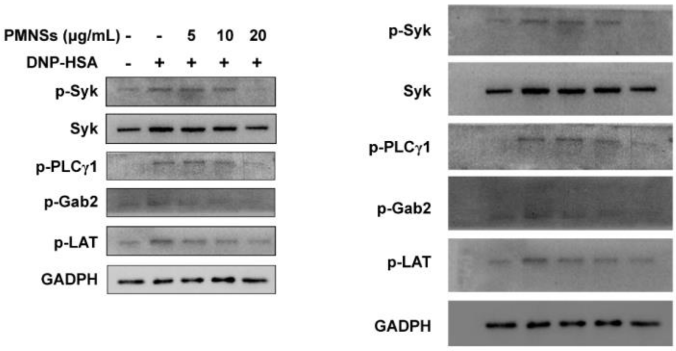

Figure S16. The respective western blot images presented in Figure 2e and their corresponding original images.

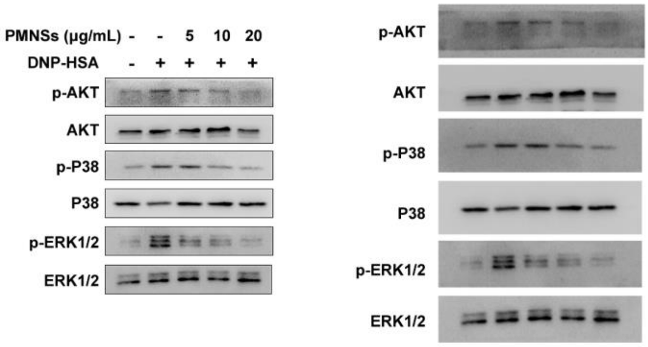

Figure S17. The respective western blot images presented in Figure 2g and their corresponding original images.

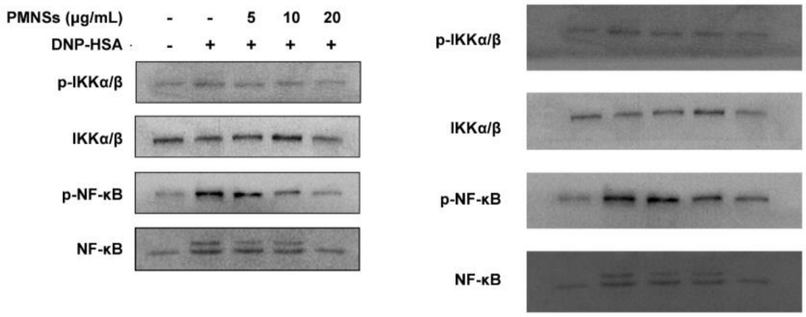

Figure S18. The respective western blot images presented in Figure S12 and their corresponding original images.
